# Association of diabetes and obesity with sperm parameters and testosterone levels: a meta-analysis

**DOI:** 10.1186/s13098-021-00728-2

**Published:** 2021-10-16

**Authors:** Ou Zhong, Lin Ji, Jinyuan Wang, Xiaocan Lei, Hua Huang

**Affiliations:** 1 Reproductive Hospital of Guangxi Zhuang Autonomous Region, 530021 Nanning, China; 2 Clinical Anatomy and Reproductive Medicine Application Institute, Hengyang Medical School, University of South China, 421001 Hengyang, China

**Keywords:** Meta-analysis, Diabetes, Obesity, Sperm, Semen, Testosterone levels

## Abstract

**Background:**

The present study performed two distinct meta-analyses with common outcomes (sperm parameters); one was performed in obese individuals (and non-obese controls) and the other in diabetic individuals (and non-diabetic controls).

**Methods:**

PubMed, Embase, The Cochrane library, Web of Science, Scopus databases were searched to collect clinical studies related to the effects of obesity and diabetes on male sperm from inception to on 1st February 2021. Statistical meta-analyses were performed using the RevMan 5.4 software. Stata16 software was used to detect publication bias. The methodological quality of the included studies was assessed with the Ottawa–Newcastle scale using a star-based system.

**Results:**

A total of 44 studies were finally included in the present study, which enrolled 20,367 obese patients and 1386 patients with diabetes. The meta-analysis results showed that both obesity and diabetes were associated with reduced semen volume (obese versus non-obese controls: mean difference (MD) = − 0.25, 95% CI = (− 0.33, − 0.16), p < 0.001; diabetes versus non-diabetic controls: MD = − 0.45, 95% CI = (− 0.63, − 0.27), p < 0.001), reduced sperm count (obese versus non-obese controls: MD = − 23.84, 95% CI = (− 30.36, − 17.33), p < 0.001; diabetes versus non-diabetic controls: MD = − 13.12, 95% CI = (− 18.43, − 7.82), p < 0.001), reduced sperm concentration (obese versus non-obese controls: MD = − 7.26, 95% CI = (− 10.07, − 4.46), p < 0.001; diabetes versus non-diabetic controls: MD = − 11.73, 95% CI = (− 21.44, − 2.01), p = 0.02), reduced progressive motility (obese versus non-obese controls: MD = − 5.68, 95% CI = (− 8.79, − 2.56), p < 0.001; diabetes versus non-diabetic controls: MD = − 14.37, 95% CI = (− 21.79, − 6.96), p = 0.001), and decreased testosterone levels (obese versus non-obese controls: MD = − 1.11, 95% CI = (− 1.92, − 0.30), p = 0.007; diabetes versus non-diabetic controls: MD = − 0.37, 95% CI = (− 0.63, − 0.12), p = 0.004).

**Conclusions:**

Current evidence suggests that obesity and diabetes negatively affect sperm parameters in men and are associated with low testosterone levels. Due to the limitation of the number and quality of included studies, the above conclusions need to be verified by more high-quality studies.

**Supplementary Information:**

The online version contains supplementary material available at 10.1186/s13098-021-00728-2.

## Background

Infertility is a serious health problem that occurs in approximately 10% of all couples worldwide [1]. Diabetes mellitus, obesity, environmental factors, genetic and epigenetic factors contribute to infertility. Abnormal sperm parameters cause infertility in approximately 50% of couples without children [2]. Obesity and diabetes are fast growing health problems, approximately 463 million people were affected by diabetes and 1.9 billion people are overweight (body mass index (BMI) ≥25 kg/m^2^) or affected by obesity (BMI ≥ 30 kg/m^2^) in the world [3–6]. Studies have shown that obesity or diabetes mellitus is associated with decreased sperm quality [2, 7], the possible mechanism underlying diminished reproductive performance in those patients is representing an altered hypothalamic–pituitary–gonadal axis, peripheral aromatization of steroids to oestrogen, with decreased testosterone, increased oestradiol levels [8]. Spermatogenesis and sperm quality depend on high levels of intratesticular testosterone. A clinical study showed that low testosterone level is associated with poor semen total motility, progressive motility, and morphology [9], but the testosterone level increased and the sperm quality improved after the Letrozole (an aromatase inhibitor) treatment [10].

The effect of diabetes and obesity on sperm count, motility, and morphology in humans is controversial. Some studies have shown that obesity and diabetes do not affect sperm quality compared with controls [11, 12]. Keskin et al., found that BMI had no effects on semen parameters although it exhibited negative correlation with prolactin and testosterone levels [13]. Ghsemi et al., demonstrated that zinc and magnesium levels in seminal plasma of nondiabetic men were more elevated than in diabetic groups. Znic showed positive and significant correlations with sperm motility and morphology. Magnesium was significantly correlated with sperm motility and morphology [14]. Karimi et al., showed that men with diabetes had significantly higher mean levels of receptor for advanced glycation end products protein and DNA fragmentation in spermatozoa [7]. Due to the controversies among different studies, the present study includes two distinct meta-analyses with common outcomes (sperm parameters); one was performed in obese individuals (and controls) and the other in diabetic individuals (and controls). The aim of this study was to assess the effect of obesity and diabetes on semen parameters. We furthermore investigated whether the state of obesity and diabetes directly affects the reproductive potential of men by causing abnormal testosterone levels, which is considered to be one of the causes of infertility in men.

## Materials and methods

### Search strategy

PubMed, Embase, The Cochrane library, Web of Science, Scopus databases were searched to collect clinical studies (case-control studies) related to the effects of obesity and diabetes on male sperm. The search was carried out by combining subject words and free words. See Additional file 1: Annex S1 for detailed search words. The latest search was performed on 1st February 2021.

### Inclusion and exclusion criteria

The inclusion criteria were as follows: (1) obesity: BMI ≥ 30 kg/m^2^, control: BMI < 25 kg/m^2^, patients with any disorders of the reproductive system were excluded from the study; (2) diabetes: men diagnosed with diabetes (type 1 or type 2 diabetes); control group: healthy men without diabetes (Table 1); patients with any disorders of the reproductive system were excluded from the study; (3) types of studies included: case-control studies; (4) Literature published in English. The exclusion criteria were as followed: (1) case-series/reports, expert opinions, basic science, conference abstracts and review articles; (2) Animal experiments, cell experiments and other articles without available data; (3) articles with poor quality and obvious statistical errors.

**Table.1:** Characteristics of people with diabetes included in the study

Study	Type of study	Duration of the disease	Type of diabetes	Inclusion criteria
Imani et al., (2020)	Case–control	–	Type 2	Men with type diabetes and healthy controls
Lu et al., (2017)	Case–control	33.29 (20–46) months	–	Men with diabetes and healthy controls
Ghasemi et al., (2016)	Case–control	> 5 years	Type 1, n = 15 Type 2, n = 10	Men with diabetes and healthy controls
Bhattacharya et al., (2014)	Case–control	–	–	Men without history of epididymo-orchitis; hernia/hydrocoele or history of surgery; chemotherapy for any malignancy; varicocoele, testicular growth, ejaculatory duct obstruction, and primary spermatogenic defects
Singh et al., (2014)	Case–control	–	Type 2	Men with type 2 diabetes and healthy controls
Verit et al., (2014)	Case–control	–	Insulin resistance	The patients over 40 years, with known erectile dysfunction, chronic/hereditary disease (including prostatitis, hypertension, dyslipidemia needing medical care), malignancy, and smokers, alcohol drinkers, drug abusers, azoospermics and patients who had varicocele were excluded
Rama Raju et al., (2012)	Case–control	3.95 ± 3.07 years	Type 2	Inclusion criteria for the diabetic group included patients with value of HbA1c≥7.0 % and those with a value of ≤5.7 % were included in the nondiabetic group Patients with a history of drug or alcohol abuse, heavy cigarette smoking, hypergonadotrophic hypogonadism and leukocytospermia were excluded from the study Patients who underwent assisted reproductive procedures involving cryopreserved and testicular extracted sperm samples were also excluded from the study
Karimi et al., (2012)	Case–control	>5 years	Type 1, n = 17 Type 2, n = 15	Men with diabetes and healthy controls
Agbaje et al., (2007)	Case–control	–	Type 1	Men with type 1 diabetes and healthy controls
Baccetti et al., (2002)	Case–control	11.3 ± 8.0 years	Type 1	Men with hypergonadotrophic hypogonadism were excluded No patients had signs of neuropathy or were impotent Men with diabetes free of renal disease, haemochromatosis, or any medication other than insulin were selected Exclusion criteria were history of drug or alcohol abuse, ongoing medical treatment with anabolic steroids and gonadotrophins, heavy smoking habit (10> cigarettes/day), hypertension, leukocytospermia, varicocele and unilateral testicular atrophy
Ali et al., (1993)	Case–control	Mean 11.2 years	Type 1, n = 100 Type 2, n = 314	Men with insulin-dependent (IDDM) diabetes and age-matched nondiabetic controls men with non-insulin-dependent (NIDDM) diabetes and age-matched nondiabetic controls All the people with diabetes were otherwise healthy with variable diabetic complications and no other medical diseases
García-Díezet al., (1991)	Case–control	–	Type 1	Men with type 1 diabetes and healthy controls
Murray et al., (1988)	Case–control	14.9 ± 3.3 years	Type 1	People with diabetes were drawn from a population with a recent history of stable glycemic control. One subject also had Addison’s disease. Two patients with diabetes had background retinopathy, one had proliferative retinopathy, and three patients had mild peripheral neuropathy restricted to the feet. One patient had proteinuria greater than 2 g/24 hrs but a normal serum creatinin
Padrón et al., (1984)	Case–control	11.6 (2-21) years	Type 1	Adolescents with type 1 diabetes and healthy controls

### Literature screening and data extraction

After literature retrieval, at least two members independently screened the title, abstract and full text of the articles according to the inclusion and exclusion criteria. Duplicate articles were eliminated first, and then titles and abstracts were read. After the articles with obviously unrelated contents were excluded, the full text was further read to determine whether the included articles were included or not, and then data extraction was conducted for the included articles.

### Quality assessment

The methodological quality of the included studies was assessed with the Ottawa–Newcastle scale using a star-based system, which evaluates the selection of the study groups, the comparability of the groups, and the ascertainment of the outcome of interest [15]. Studies that scored > 7 were considered to have a low risk of bias, scores of 5–7 were considered as having moderate risk of bias, and scores of < 5 were indicative of a high risk of bias.

### Indicators

We defined as primary outcomes the semen volume (ml), the total sperm count (million), the sperm concentration (million/ml) and progressive motility (%). We complementarily investigated the serum levels of testosterone (in ng/ml or nmol/l).

### Statistical analysis

Statistical meta-analyses were performed using the RevMan 5.4 software. Confidence intervals (CIs) were set at 95%. Continuous data were calculated with weighted mean difference (MD), and confidence intervals were set at 95%, p < 0.05 was considered statistically significant. Testosterone was calculated using Standardized Mean Difference (SMD) because of the different data units. Heterogeneity between studies was evaluated by I^2^ and p values. If I^2^ > 50 % and p < 0.1, heterogeneity across studies was considered to be existed, and the SMD and MD was calculated using the random-effects model; otherwise, the fixed-effects model was applied. SMD and MD for all primary and secondary outcomes were calculated, using the random effect model due to the significant heterogeneity in the included studies. Stata16 software was used to detect publication bias, Egger and Begg methods were mainly used, P > 0.05 indicates no significant publication bias (because Egger examination is more sensitive, when the two results are contradictory, the Egger examination results are given priority).

## Results

### Study selection

**Fig. 1:**
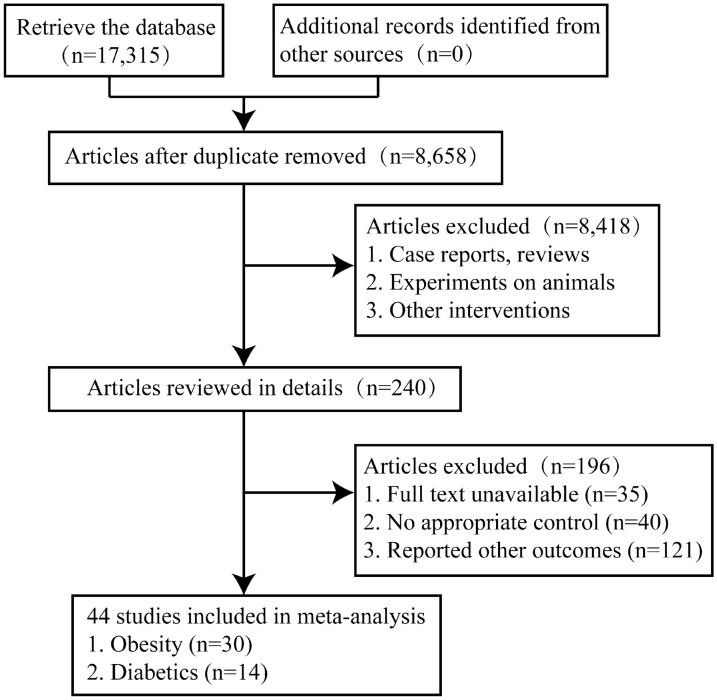
Flowchart of study selection

We identified 17,315 articles in the initial retrieval, including PubMed (n = 3587), Embase (n = 6877), The Cochrane library (n = 705), Web of Science (n = 1943) and Scopus (n = 4203). Of these, 8657 duplicate articles were excluded after carefully examining the titles and abstracts. After further screening, 44 studies were included in the meta-analysis, the literature screening process and results were shown in Fig. 1.

### Study characteristics

Forty-four studies were finally included in the present study, which enrolled 20,367 obese patients and 1386 people with diabetes. The Ottawa-Newcastle scale was used to evaluate the quality of the included articles, and the results showed that all the 44 included studies reached a medium or high level. However, some of the studies did not describe whether continuous cases were included, and none of the included studies described whether blind method was used, and thus, the blindness criteria was not included in the Ottawa-Newcastle scale. The demographic data of the patients are shown in Table 2.

**Table 2:** Demographic of the patients in the study

Study	Country	Population size (cases/controls)	Age, year (cases/controls)	BMI, kg/m^2^ (cases/controls)	Newcastle Ottawa
*Obesity*					
Oztekin, 2020 [2]	Turkey	62/146	31.2 ± 5.5/30.2 ± 5.2	33.4 ± 2.9/22.6 ± 1.7	8
Salas-Huetos, 2020 (1) [32]	USA	12/12	23.6 ± 0.3/23.5 ± 0.3	36.7 ± 2.3/20.4 ± 0.4	8
Salas-Huetos,A.2020 (2) [32]	USA	12/12	30.5 ± 0.1/30.3 ± 0.1	34.2 ± 0.9/21.9 ± 0.5	8
Salas-Huetos 2020 (3) [32]	USA	12/12	40.5 ± 0.4/40.8 ± 0.1	37.6 ± 2.0/23.2 ± 0.3	8
Pini, 2020 [33]	USA	5/5	41.0 ± 2.1/38.2 ± 2.2	33.0 ± 0.6/23.9 ± 0.4	6
Abbasihormozi, 2019 [22]	Iran	40/40	33 ± 0.97/33 ± 0.97	36 ± 0.80/23.3 ± 0.21	8
Chen, 2019 [34]	China	28/143	37.25 ± 7.8/35.98 ± 8.92		7
Taha, 2019 [35]	Egypt	96/92	35 ± 6.54/36.5 ± 7.1	-	7
Ferigolo, 2019 [36]	Brazil	27/20	-	36.9 ± 8.22/23.2 ± 1.48	6
Calderón, 2019 [37]	Spain	20/10	40 ± 8/34 ± 5	48 ± 9/24 ± 2	7
Qi, 2018 [38]	China	27/28	–	–	5
Ramaraju, 2018 [39]	India	201/437	35.2 ± 4.4/33.9 ± 4.7	–	8
Oliveira, 2017 [40]	Brazil	598/370	38.0 ± 6.4/38.3 ± 7.0	–	7
Engin-Ustun, 2018 [17]	Turkey	53/53	33.32 ± 6.64/32.21 ± 5.82	–	8
Wang, 2017 [41]	China	298/1398	32.9 ± 1.8/32.1 ± 2.0	–	7
Luque, 2017 [42]	Argentina	468/747	36.4 ± 0.2/34.9 ± 0.2	32.6 ± 0.1/23.6 ± 0.1	8
Keskin, 2017 [13]	Turkey	56/165	–	33.09 ± 3.44/22.65 ± 1.69	6
Taha, 2016 [8]	Egypt	25/81	38.5 ± 5.3/36.0 ± 4.7	– 32.7 ± 2.5/21.7 ± 1.7	7
Alshahrani, 2016 [43]	Saudi Arabia	185/75	37.37 ± 6.69/35.64 ± 6.56	34.88 ± 5.31/23.05 ± 1.34	7
Garolla, 2015 [44]	Italy	20/20	37.5 ± 9.1/34.2 ± 8.6	35.8 ± 4.0/23.5 ± 5.0	8
Samavat, 2014 [45]	Italy	23/25	39.6 ± 10.7/39.2 ± 6.2	44.3 ± 5.9/24.2 ± 1.0	8
Shuangyong, 2014 [46]	–	59/58		–	3
Leisegang, 2014 [47]	South Africa	23/19	37.9 ± 7.3/35.1 ± 5.9	35.8 ± 4.3/25.5 ± 2.4	8
Belloc, 2014 [48]	France	634/5799	38.0 ± 7.3/36.4 ± 6.4	–	6
La Vignera, 2012 [49]	Italy	50/50	31.5 ± 1.1/31.6 ± 1.7	–	8
Fariello, 2012 [50]	Brazil	36/82	34.3 ± 4.9/33.5 ± 6.1	–	5
Rybar, 2011 [11]	Czech Republic	16/74	32.5 ± 4.0/30.2 ± 5.9	–	6
Belloc, 2011 [51]	–	400/3415	–	–	3
Shayeb, 2011 [52]	UK	269/839	34.0 ± 5.8/32.4 ± 6.0	–	7
Paasch, 2010 [53]	Germany	245/1003	34.3 ± 0.56/27.8 ± 0.26	32.7 ± 0.19/22.5 ± 0.04	8
Martini, 2010 [54]	Argentina	155/251	36.0 ± 0.5/34.1 ± 0.4	33.2 ± 0.3/23.4 ± 0.1	7
Qin, 2007 [55]	China	17/690	39.0 ± 9.9/38.4 ± 9.9	31.4 ± 1.6/22.2 ± 1.8	8
*Diabetics*					
Imani, 2020 [56]	Iran	30/30	33.5 ± 1.1/34.1 ± 1.5	25.51 ± 1.69/24.75 ± 1.15	7
Lu, 2017 [57]	China	30/30	Aged 21–49 years	–	8
Ghasemi, 2016 [14]	Iran	25/25	Aged 22–46 years	–	7
Singh, 2014 [58]	India	25/25	47.8 ± 3.0/44.3 ± 2.3	–	5
Verit, 2014 [59]	Turkey	40/40	31.2 ± 5.0/29.6 ± 5.0	25.6 ± 3.3/25.0 ± 3.3	8
Bhattacharya, 2014 [60]	India	52/66	36.29 ± 5.29/34.92 ± 4.58	27.68 ± 3.88/27.57 ± 3.83	7
Rama Raju, 2012 [61]	India	24/52	–	–	8
Karimi, 2012 [7]	Iran	32/35	35.84 ± 8.89/32.58 ± 5.68	–	7
Agbaje, 2007 [4]	UK	27/29	34.0 ± 2.0/32.7 ± 0.7	–	6
Baccetti, 2002 [12]	Italy	22/24	38 ± 6/37 ± 5	26 ± 4/27 ± 3	8
Ali, 1993 (1) [62]	Japan	100/100	Mean 54 years	–	6
Ali, 1993 (2) [62]	Japan	314/100	Mean 54 years	–	6
García-Díez, 1991 [63]	Spain	7/10	–	–	4
Murray, 1988 [64]	USA	8/10	23 ± 0.8/26 ± 1.7	23.2 ± 1.50/22.4 ± 1.24	5
Padrón, 1984 [65]	Spain	32/42	Mean 18.6 years (range 17 to 22 years)	–	4

### Effects of obesity and diabetes on semen volume

**Fig. 2:**
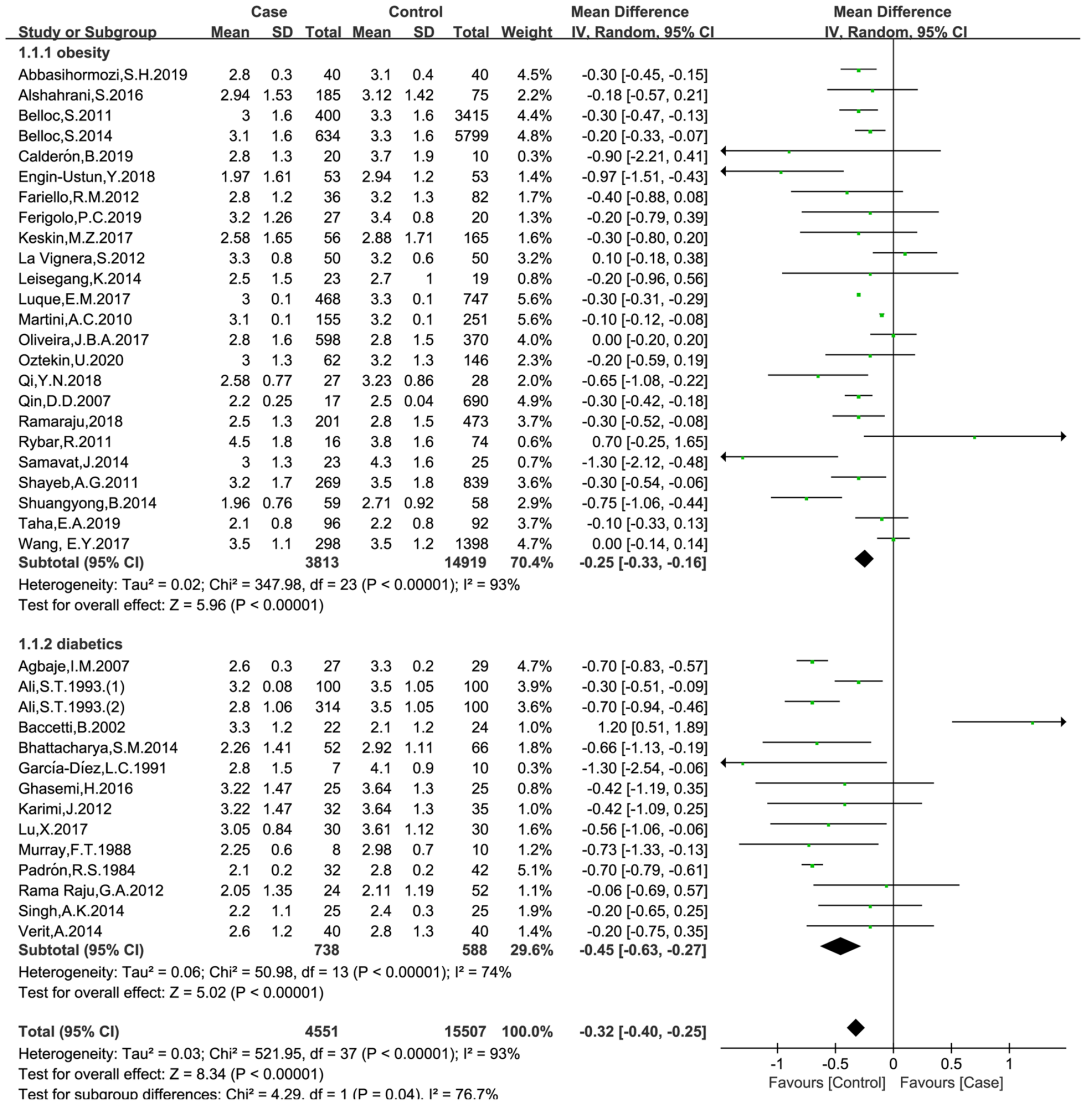
Meta-analysis of the effects of obesity and diabetes on semen volume

The data were available in 37 trials, including 4551 patients in the case group and 15,507 patients in the control group. The random effects model was used for analyses. The results of meta-analysis showed that both obesity and diabetes were associated reduced semen volume (obese versus non-obese controls: MD = − 0.25, 95% CI = (− 0.33, 0.16), p < 0.001; diabetes versus non-diabetic controls: MD = − 0.45, 95% CI = (− 0.63, − 0.27), p < 0.001; Fig. 2). No significant publication bias was found in the results of Begg’s plots (p = 1.9929) and Egger’s test (p = 0.1407) for semen volume.

### Effects of obesity and diabetes on total sperm count

**Fig. 3:**
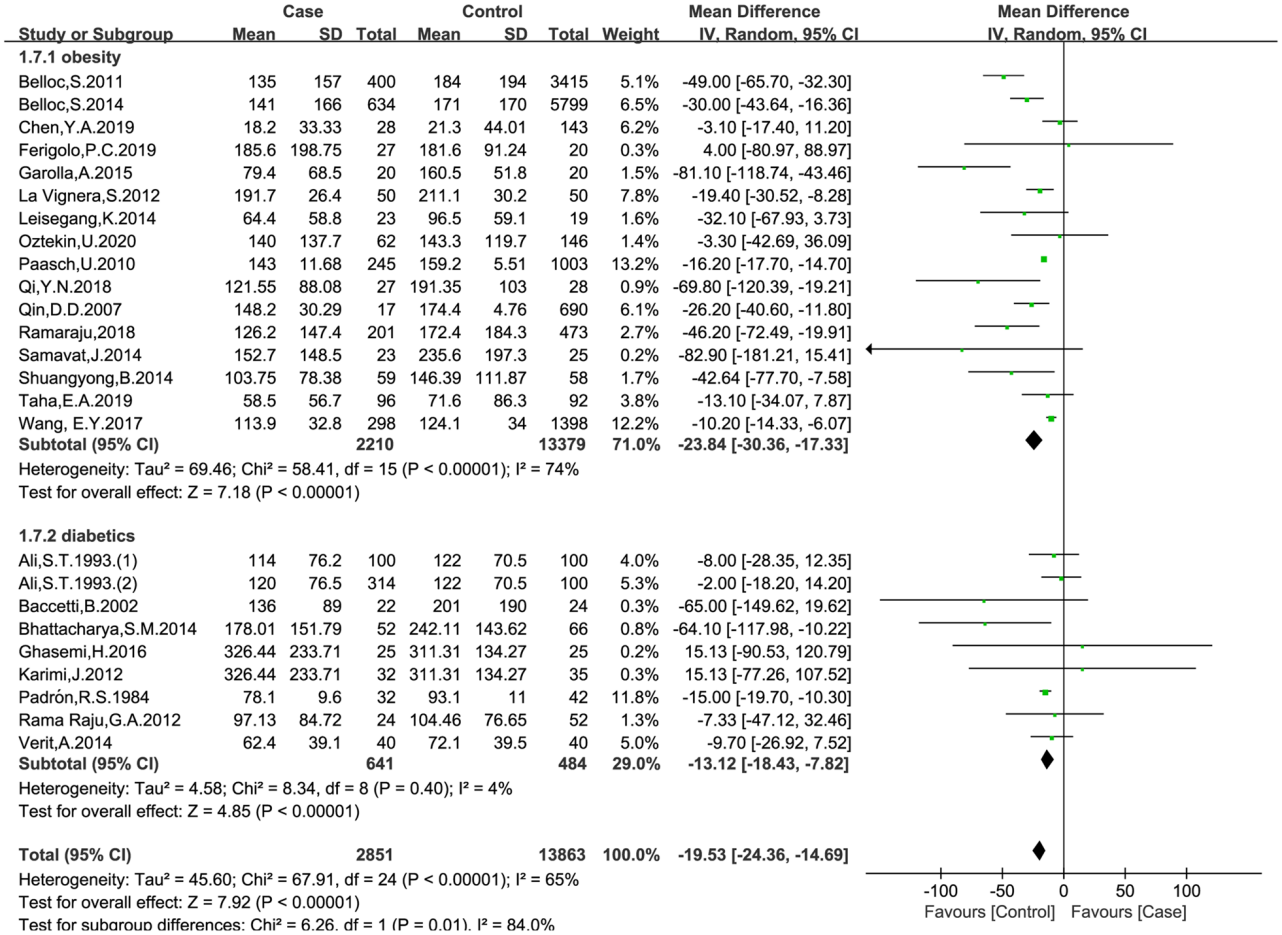
Meta-analysis of the effects of obesity and diabetes on sperm count

The data were available in 24 trials, including 2851 patients in the case group and 13,863 patients in the control group. The random effects model was used for analyses. The results of meta-analysis showed that: both obesity and diabetes were associated with reduced sperm count (obese versus non-obese controls: MD = − 23.84, 95% CI = (− 30.36, − 17.33), p < 0.001; diabetes versus non-diabetic controls: MD = − 13.12, 95% CI = (− 18.43, − 7.82), p < 0.001; Fig. 3). No significant publication bias was found in the results of Begg’s plots (p = 1.9959) and Egger’s test (p = 0.4478) for sperm count.

### Effects of obesity and diabetes on sperm concentration

**Fig. 4:**
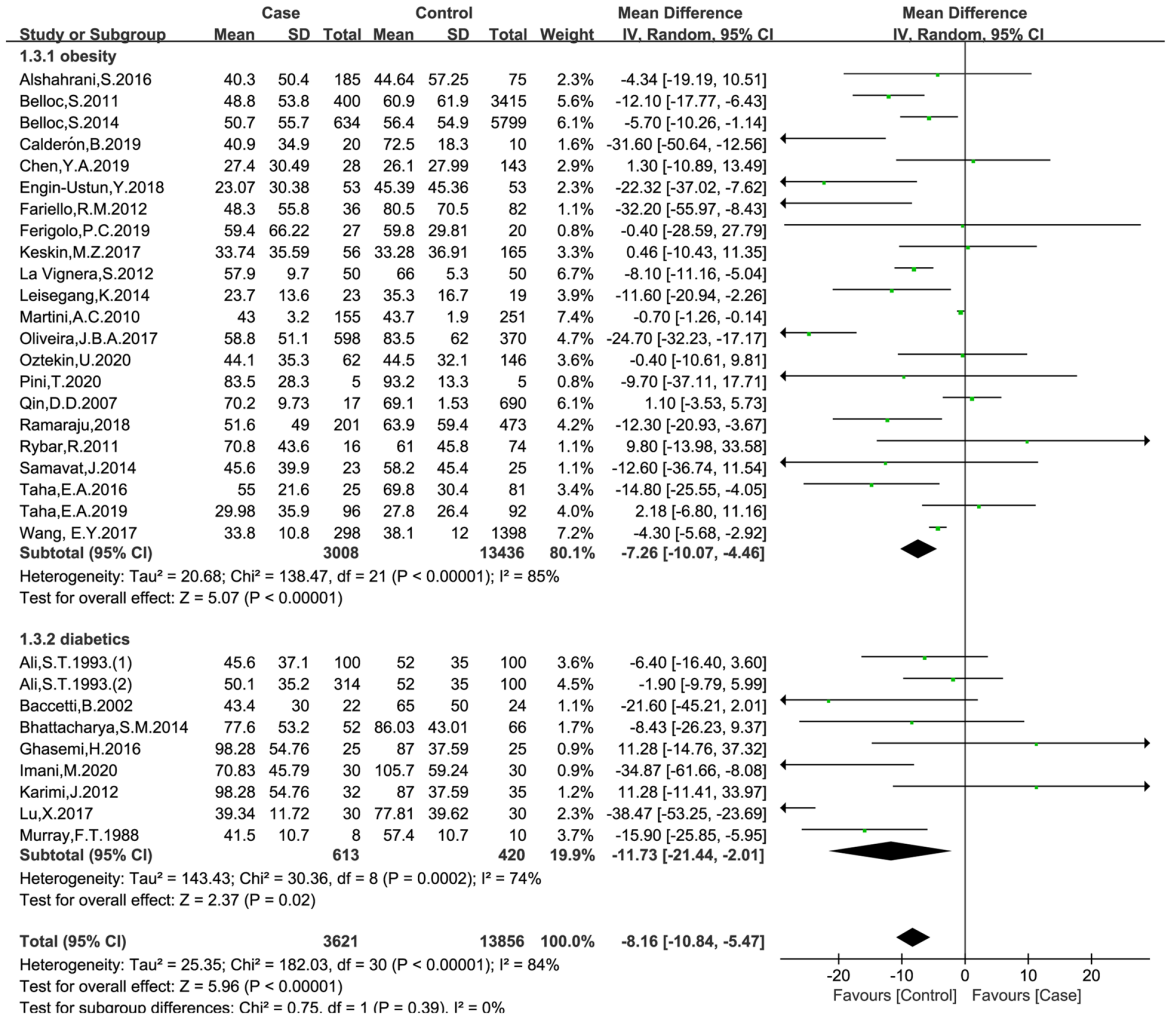
Meta-analysis of the effects of obesity and diabetes on sperm concentration

The data were available in 30 trials, including 3621 patients in the case group and 13,856 patients in the control group. The random effects model was used for analyses. The results of meta-analysis showed that both obese and diabetes were associated with reduced sperm concentration (obese versus non-obese controls: MD = − 7.26, 95% CI = (− 10.07, -4.46), p < 0.001; diabetes versus non-diabetic controls: MD = − 11.73, 95% CI = (− 21.44, − 2.01), p = 0.02; Fig. 4). No significant publication bias was found in the results of Begg’s plots (p = 1.7790) and Egger’s test (p = 0.1084) for sperm concentration.

### Effects of obesity and diabetes on progressive motility

**Fig. 5:**
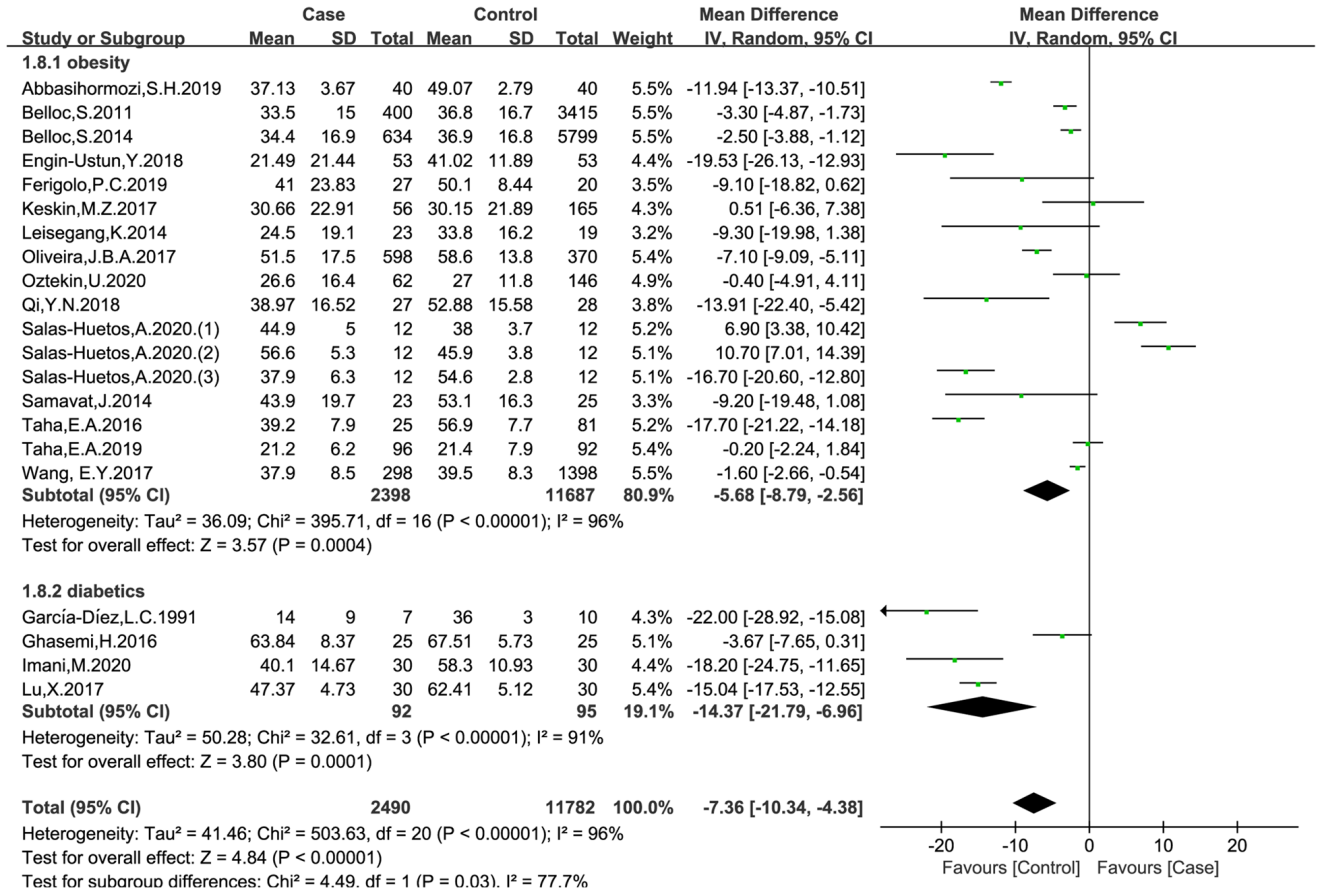
Meta-analysis of the effects of obesity and diabetes on progressive motility

The data were available in 19 trials, including 2490 patients in the case group and 11,782 patients in the control group. The random effects model was used for analyses. The results of meta-analysis showed that both obesity and diabetes were associated with progressive motility (obese versus non-obese controls: MD = − 5.68, 95% CI = (− 8.79, -2.56), p < 0.001; diabetes versus non-diabetic controls: MD = − 14.37, 95% CI (− 21.79, − 6.96), p = 0.001; Fig. 5). No significant publication bias was found in the results of Begg’s plots (p = 1.7611) and Egger’s test (p = 0.1140) for progressive motility.

### Effects of obesity and diabetes on testosterone level

**Fig. 6:**
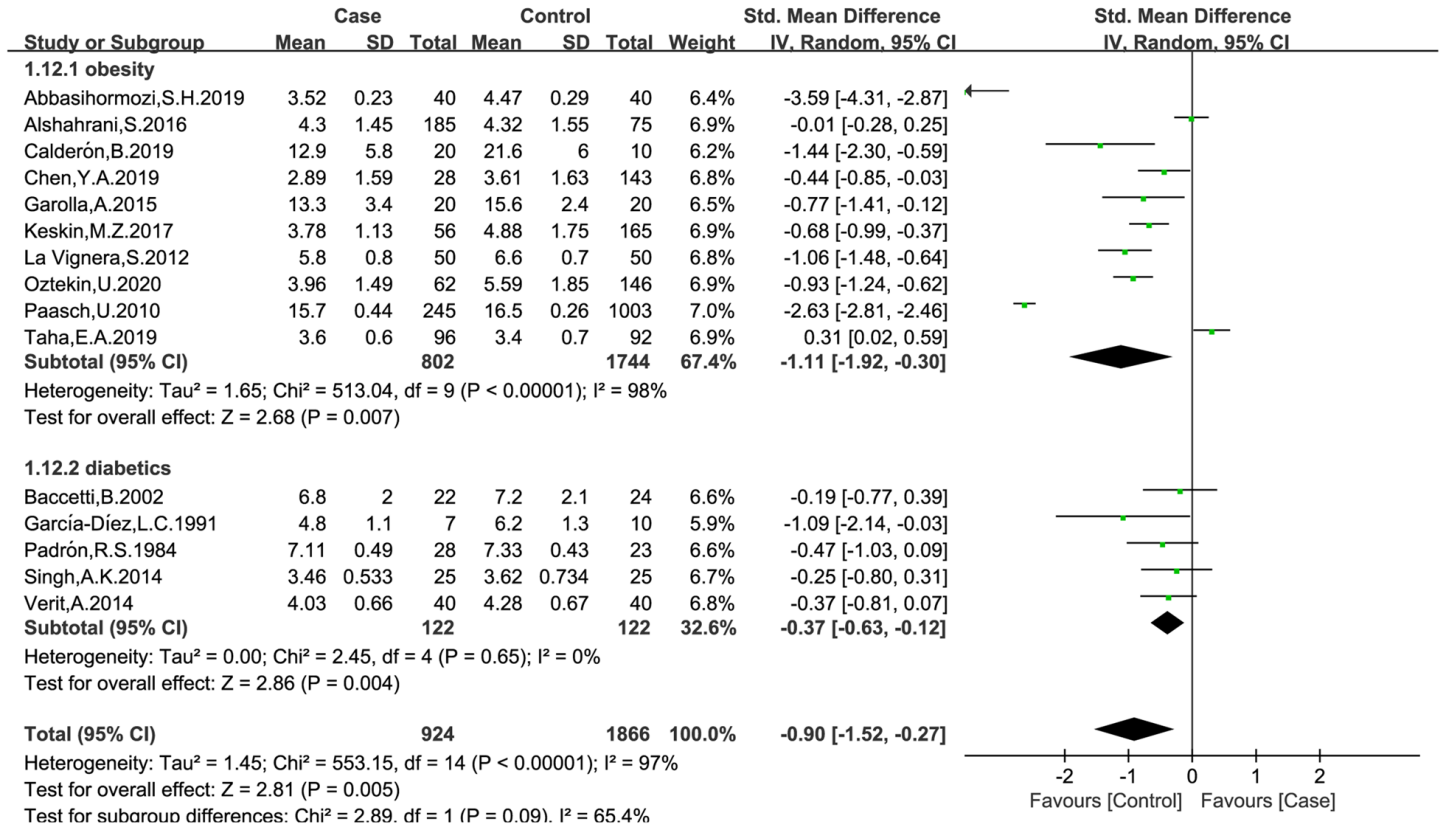
Meta-analysis of the effects of obesity and diabetes on testosterone levels

The data were available in 15 trials, including 924 patients in the case group and 1,866 patients in the control group. The random effects model was used for analyses. The results of meta-analysis showed that both obesity and diabetes were associated decreased testosterone level (obese versus non-obese controls: MD = − 1.11, 95% CI = (− 1.92, -0.30), p = 0.007; diabetes versus non-diabetic controls: MD = − 0.37, 95% CI = (− 0.63, − 0.12), p = 0.004; Fig. 6). No significant publication bias was found in the results of Begg’s plots (p = 1.9252) and Egger’s test (p = 0.4715) for testosterone.

### Subgroup analysis of effects of diabetes on sperm parameters and testosterone levels

The subgroup analysis showed that type 1 diabetes were associated with reduced semen volume (Additional file 2: Figure S1); reduced semen count (Additional file 2: Figure S2); reduced semen concentration (Additional file 2: Figure S3); reduced progressive motility (Additional file 2: Figure S4), but not testosterone levels (Additional file 2: Figure S5). Furthermore, type 2 diabetes were associated with reduced progressive motility (Additional file 2: Figure S3), but not the other parameters including semen volume (Additional file 2: Figure S1), semen count (Additional file 2: Figure S2), semen concentration (Additional file 2: Figure S3) or testosterone levels (Additional file 2: Figure S5). The mixed and unclear type of diabetes were associated with reduced semen volume (Additional file 2: Figure S1), but not the other parameters including semen count (Additional file 2: Figure S2), progressive motility (Additional file 2: Figure S4) or testosterone levels (Additional file 2: Figure S5).

## Discussion

In the present study, we included 44 studies, which enrolled 20,367 obese patients and 1386 patients with diabetes. The meta-analysis results showed that both obesity and diabetes were associated with reduced semen volume, reduced sperm count, reduced sperm concentration, reduced progressive motility, and decreased testosterone levels. Our results indicate that obesity and diabetes negatively affect sperm parameters in men and are associated with low testosterone levels.

Studies have shown that obesity could affect sperm quality and reproductive potential, alters the regional microenvironment of spermatogenesis in testis and sperm maturation in epididymis, and finally results in poor sperm quality [16]. High BMI (> 30 kg/m^2^) are associated with decrease in sperm concentrations and total sperm counts compared with BMI < 25 kg/m^2^ [17].These alterations in spermatogenesis have also been evidenced in animal models, for example, a mouse model of obesity from a high-fat diet alters the metabolic status and sperm function of F0 mice [18]. The decrease of reproductive function in obese patients may be related to the following reasons: (1) Obesity can lead to endocrine disorders and affect the regulation function of the hypothalamic-pituitary-testicular axis. A large cohort study has shown that the increase of BMI has negative effects on luteinizing hormone, testosterone, and statin B in men [19, 20]. (2) Obesity can increase the temperature of scrotum, but the optimal temperature for sperm growth is 34 ~ 35 ℃. In obese patients, the local temperature of scrotum increases due to thicker scrotal fat, thus affecting semen quality. The study of Yaeram et al. pointed out that increased scrotal temperature would lead to lower sperm count and testicular weight in mice, affecting sperm quality [21]. (3) Obesity can aggravate sperm DNA damage. Abbasihormozi et al. analyzed the semen of 40 obese men and 40 healthy men, and the results showed that the levels of reactive oxygen species (ROS) and the increase of DNA fragments in the sperm of obese men were significantly higher than that of normal healthy men [22].

Recent studies have shown that diabetes has adverse effects on male sexual and reproductive function, including impaired spermatogenesis, reduced serum testosterone level and semen volume, resulting in low libido and erectile dysfunction, as well as ejaculation difficulties and infertility [23]. PCNA is an intraconuclear polypeptide, which plays an important role in the regulation of cell cycle. It promotes mitosis of spermatogonia and meiosis of spermatocyte, thus accelerating the generation of sperm. Tanaka et al. believed that PCNA could reflect the spermatogenic function of testis in male infertility patients to some extent [24]. Studies have shown that diabetes has a negative effect on the expression of PCNA in testicular tissue, and with the progression of diabetes (the increase of blood glucose), PCNA expression shows a significant downward trend, which damages the function of testis [25]. Cameron et al. demonstrated that low testosterone levels and abnormal sperm count morphology in rats with diabetes were associated with high blood glucose levels [26]. Patients with diabetes are in a physiological state of hyperglycemia for a long time, which activates the oxidative stress response of the body and causes vascular endothelial injury, resulting in structural abnormalities of the testis and epididymis. At the same time, high blood glucose can affect the regulation function of the hypothalamic-pituitary-gonadal axis, and cause changes in the number and morphology of testicular interstitial cells as well as degeneration of sertoli cells, leading to decreased testosterone synthesis and secretion ability, impeding the occurrence and maturation of sperm, and thus affecting reproductive function [27].

The results of this study show that both obesity and diabetes can reduce semen volume, reduce sperm count, reduce sperm concentration, reduce progressive motility, and decrease testosterone levels. Pergialiotis et al.‘s meta-study on diabetes also showed the same results, but some statistical data were wrong in this study. For example, Handelsman et al., sued Mean ± SEM to present the data [28], while the authors did not convert the data and directly integrated the results into Mean ± SD for analysis [29]. A meta-analysis by Pergialiotis et al., showed that diabetes seems to decrease the seminal volume and the percentage of motile cells, and increase the follicle-stimulating hormone values of men who were screened for infertility; while diabetes also influenced the total sperm count, the percentage of normal sperm morphology, or luteinizing Hormone and testosterone values [29]. A meta-analysis by Sermondade et al., showed that overweight and obesity were associated with an increased prevalence of azoospermia or oligozoospermia [30]. Wang et al., selected ordinary obese men rather than infertile patients to conduct a meta-analysis, and the results showed that obesity had no effect on sperm concentration and percentage of normal sperm morphology, but decreased semen volume, total sperm number, percentage of forward progression and percentage of viability [31], suggesting that obesity affects semen quality to a certain extent, and maintaining normal weight may be one of the effective ways to improve male fertility. The present further included the most updated studies to perform the meta-analysis and demonstrated that obesity and diabetes could both influence the sperm parameters.

The limitations of this systematic evaluation were as follows: (1) The types of people with diabetes included were different, some were type 1 diabetes and some were type 2 diabetes; (2) The large difference in the sample size of the included studies may cause some heterogeneity; (3) Only English literature was included in this study, which may affect the extrapolation of the results; (4) Some studies did not describe whether continuous cases were included or not, and none of the included studies described whether blind method was used.

## Conclusions

In conclusion, current evidence suggests that obesity and diabetes negatively affect sperm parameters in men and are associated with low testosterone levels. Due to the limitation of the number and quality of included studies, the above conclusions need to be verified by more high-quality studies.

## Supplementary Information

**Additional file 1.** Appendix for literature search stratedgy.

**Additional file 2.** Sub-group meta-analysis based on the tyeps of diabetes.

## Data Availability

All the data are available upon reasonable request.
