# Supplementary material for: Association of diabetes and obesity with sperm parameters and testosterone levels: a meta-analysis
Source: Diabetol Metab Syndr. 2021 Oct 16;13:109. doi: 10.1186/s13098-021-00728-2 (PMC8520257; doi:10.1186/s13098-021-00728-2)
Supplement: Supplementary file 1 — Additional file 1. Appendix for literature search stratedgy. [file 13098_2021_728_MOESM1_ESM.docx]

**17,315**

**Appendix 1.Pubmed search strategy**

Searched February 6, 2021

#1 "Diabetes Mellitus"[MeSH Terms] OR "Diabetes Complications"[MeSH Terms] (436,362)

#2 "diabet*"[Title/Abstract] (661,710)

#3 "IDDM"[Title/Abstract] OR "NIDDM"[Title/Abstract] OR "MODY"[Title/Abstract] OR "T1DM"[Title/Abstract] OR "T2DM"[Title/Abstract] OR "T1D"[Title/Abstract] OR "T2D"[Title/Abstract] (57,956)

#4 ("insulin*"[Title/Abstract] OR "noninsulin*"[Title/Abstract]) AND "depend*"[Title/Abstract] (73,302)

#5 "insulinodepend*"[Title/Abstract] OR "noninsulinodepend*"[Title/Abstract] (33)

#6 #1 OR #2 OR #3 OR #4 OR #5 (751,898)

#7 "Obesity"[MeSH Terms] OR "Overweight"[MeSH Terms] OR "Weight Gain"[MeSH Terms] OR "Body Mass Index"[MeSH Terms] (325,262)

#8 "obes*"[Title/Abstract] OR "adipos*"[Title/Abstract] OR "Overweight"[Title/Abstract] OR "Over weight"[Title/Abstract] OR "weight gain"[Title/Abstract] OR "Body Mass Index"[Title/Abstract] OR "BMI"[Title/Abstract] (606,887)

#9 #7 OR #8(675,577)

#10 #6 OR #9(1,274,205)

#11 "Spermatozoa"[MeSH Terms] OR "Spermatids"[MeSH Terms] OR "Spermatogonia"[MeSH Terms] OR "Sperm Count"[MeSH Terms] OR "Sperm Maturation"[MeSH Terms] OR "Sperm Motility"[MeSH Terms] OR "Oligospermia"[MeSH Terms] OR "Asthenozoospermia"[MeSH Terms] OR "Azoospermia"[MeSH Terms] (81,023)

#12 "sperm*"[Title/Abstract] OR "Semen"[Title/Abstract] (152,748)

#13 #11 OR #12 (164,413)

#14 #10 AND #13(3,587)

("Diabetes Mellitus"[MeSH Terms] OR "Diabetes Complications"[MeSH Terms] OR "diabet*"[Title/Abstract] OR ("IDDM"[Title/Abstract] OR "NIDDM"[Title/Abstract] OR "MODY"[Title/Abstract] OR "T1DM"[Title/Abstract] OR "T2DM"[Title/Abstract] OR "T1D"[Title/Abstract] OR "T2D"[Title/Abstract]) OR (("insulin*"[Title/Abstract] OR "noninsulin*"[Title/Abstract]) AND "depend*"[Title/Abstract]) OR ("insulinodepend*"[Title/Abstract] OR "noninsulinodepend*"[Title/Abstract]) OR ("Obesity"[MeSH Terms] OR "Overweight"[MeSH Terms] OR "Weight Gain"[MeSH Terms] OR "Body Mass Index"[MeSH Terms] OR ("obes*"[Title/Abstract] OR "adipos*"[Title/Abstract] OR "Overweight"[Title/Abstract] OR "Over weight"[Title/Abstract] OR "Weight Gain"[Title/Abstract] OR "Body Mass Index"[Title/Abstract] OR "BMI"[Title/Abstract]))) AND ("Spermatozoa"[MeSH Terms] OR "Spermatids"[MeSH Terms] OR "Spermatogonia"[MeSH Terms] OR "Sperm Count"[MeSH Terms] OR "Sperm Maturation"[MeSH Terms] OR "Sperm Motility"[MeSH Terms] OR "Oligospermia"[MeSH Terms] OR "Asthenozoospermia"[MeSH Terms] OR "Azoospermia"[MeSH Terms] OR ("sperm*"[Title/Abstract] OR "Semen"[Title/Abstract]))

**Appendix 2.Embase search strategy**

Searched February 6, 2021

#1 'diabetes mellitus'/exp OR 'diabetes complications'/exp (1,043,121)

#2 diabet*:ab,ti(998,509)

#3 iddm:ab,ti OR niddm:ab,ti OR mody:ab,ti OR t1dm:ab,ti OR t2dm:ab,ti OR t1d:ab,ti OR t2d:ab,ti(98,298)

#4 (insulin*:ab,ti OR noninsulin*:ab,ti) AND depend*:ab,ti(92,088)

#5 insulinodepend*:ab,ti OR noninsulinodepend*:ab,ti(78)

#6 #1 OR #2 OR #3 OR #4 OR #5(1,251,853)

#7 'obesity'/exp OR 'overweight'/exp OR 'weight gain'/exp OR 'body mass index'/exp (932,807)

#8 obes*:ab,ti OR adipos*:ab,ti OR overweight:ab,ti OR 'over weight':ab,ti OR 'weight gain':ab,ti OR 'body mass index':ab,ti OR bmi:ab,ti (934,388)

#9 #7 OR #8 (1,187,462)

#10 #6 OR #9 (2,118,569)

#11 'spermatozoa'/exp OR 'spermatids'/exp OR 'spermatogonia'/exp OR 'sperm count'/exp OR 'sperm maturation'/exp OR 'sperm motility'/exp OR 'oligospermia'/exp OR 'asthenozoospermia'/exp OR 'azoospermia'/exp (94,167)

#12 sperm*:ab,ti OR semen:ab,ti (178,570)

#13 #11 OR #12(193,401)

#14 #10 AND #13(6,877)

**Appendix 3.The Cochrane library search strategy**

Searched February 6, 2021

#1 MeSH descriptor:[Diabetes Mellitus] explode all trees(31737)

#2 MeSH descriptor:[ Diabetes Complications] explode all trees(6993)

#3 (diabet*):ti,ab,kw(95825)

#4 (IDDM):ti,ab,kw(582)

#5 (NIDDM):ti,ab,kw(1078)

#6 (MODY):ti,ab,kw(24)

#7 (T1DM):ti,ab,kw(915)

#8 (T2DM):ti,ab,kw(6423)

#9 (T1D):ti,ab,kw(1487)

#10 (T2D):ti,ab,kw(3108)

#11 (insulinodepend*):ti,ab,kw(13)

#12 (noninsulinodepend*):ti,ab,kw(0)

#13 (insulin*):ti,ab,kw(62023)

#14 (noninsulin*):ti,ab,kw(2454)

#15 (depend*):ti,ab,kw(96423)

#16 (#13 OR #14) AND #15(24211)

#17 #1 OR #2 OR #3 OR #4 OR #5 OR #6 OR #7 OR #8 OR #9 OR #10 OR #11 OR #12 OR #16 (97993)

#18 MeSH descriptor:[Obesity] explode all trees(14007)

#19 MeSH descriptor:[Overweight] explode all trees(16637)

#20 MeSH descriptor:[Weight Gain] explode all trees(2580)

#21 MeSH descriptor:[Body Mass Index] explode all trees(10188)

#22 (obes*):ti,ab,kw(43555)

#23 (adipos*):ti,ab,kw(7830)

#24 (Overweight):ti,ab,kw(17044)

#25 (Over weight):ti,ab,kw(26164)

#26 (weight gain):ti,ab,kw(13367)

#27 (Body Mass Index):ti,ab,kw(42003)

#28 (BMI):ti,ab,kw(40657)

#29 #18 OR #19 OR #20 OR #21 OR #22 OR #23 OR #24 OR #25 OR #26 OR #27 OR #28 (116364)

#30 #17 OR #29 (188171)

#31 MeSH descriptor:[Spermatozoa] explode all trees(449)

#32 MeSH descriptor:[Spermatids] explode all trees(2)

#33 MeSH descriptor:[Spermatogonia] explode all trees(3)

#34 MeSH descriptor:[Sperm Count] explode all trees(331)

#35 MeSH descriptor:[Sperm Maturation] explode all trees(3)

#36 MeSH descriptor:[Sperm Motility] explode all trees(337)

#37 MeSH descriptor:[Oligospermia] explode all trees(208)

#38 MeSH descriptor:[Asthenozoospermia] explode all trees(56)

#39 MeSH descriptor:[Azoospermia] explode all trees(33)

#40 (sperm*):ti,ab,kw(6224)

#41 (semen):ti,ab,kw(1943)

#42 #31 OR #32 OR #33 OR #34 OR #35 OR #36 OR #37 OR #38 OR #39 OR #40 OR #41 (6836)

#43 #30 AND #42 (705)

**Appendix 4.Web of Science search strategy**

Searched February 6, 2021

#1 TS=(Diabetes Mellitus OR Diabetes Complications) OR AB=(diabet* OR IDDM OR NIDDM OR MODY OR T1DM OR T2DM OR T1D OR T2D OR insulinodepend* OR noninsulinodepend* OR ((insulin* OR noninsulin*)AND depend*)) (228,342)

#2 TS=(Obesity OR Overweight OR Weight Gain OR Body Mass Index) OR AB=(obes* OR adipos* OR Overweight OR Over weight OR weight gain OR Body Mass Index OR BMI) (355,182)

#3 #1 OR #2 (519,464)

#4 TS=(Spermatozoa OR Spermatids OR Spermatogonia OR Sperm Count OR Sperm Maturation OR Sperm Motility OR Oligospermia OR Asthenozoospermia OR Azoospermia) OR AB=(sperm* OR Semen) (40,518)

#5 #3 AND #4(1,943)

**Appendix 5.Scopus search strategy**

Searched February 6, 2021

( TITLE-ABS ( diabet* )  OR  TITLE-ABS ( iddm )  OR  TITLE-ABS ( niddm )  OR  TITLE-ABS ( mody )  OR  TITLE-ABS ( t1dm )  OR  TITLE-ABS ( t2dm )  OR  TITLE-ABS ( t1d ) OR  TITLE-ABS ( t2d )  OR  TITLE-ABS ( insulinodepend* )  OR  TITLE-ABS ( noninsulinodepend* )  OR  ( ( TITLE-ABS ( insulin* )  OR  TITLE-ABS ( noninsulin* ) )  AND  TITLE-ABS ( depend* ) ) )  AND  ( TITLE-ABS ( sperm* )  OR  TITLE-ABS ( semen ) ) (1,454)

( TITLE-ABS ( obes* )  OR  TITLE-ABS ( adipos* )  OR  TITLE-ABS ( overweight )  OR  TITLE-ABS ( “over weight” )  OR  TITLE-ABS ( “weight gain” ) OR  TITLE-ABS ( “Body Mass Index” )  OR  TITLE-ABS ( BMI ) )  AND  ( TITLE-ABS ( sperm* )  OR  TITLE-ABS ( semen ) ) (2,749)
