# Supplementary material for: Association of diabetes and obesity with sperm parameters and testosterone levels: a meta-analysis
Source: Diabetol Metab Syndr. 2021 Oct 16;13:109. doi: 10.1186/s13098-021-00728-2 (PMC8520257; doi:10.1186/s13098-021-00728-2)
Supplement: Supplementary file 2 — Additional file 2. Sub-group meta-analysis based on the tyeps of diabetes. [file 13098_2021_728_MOESM2_ESM.docx]

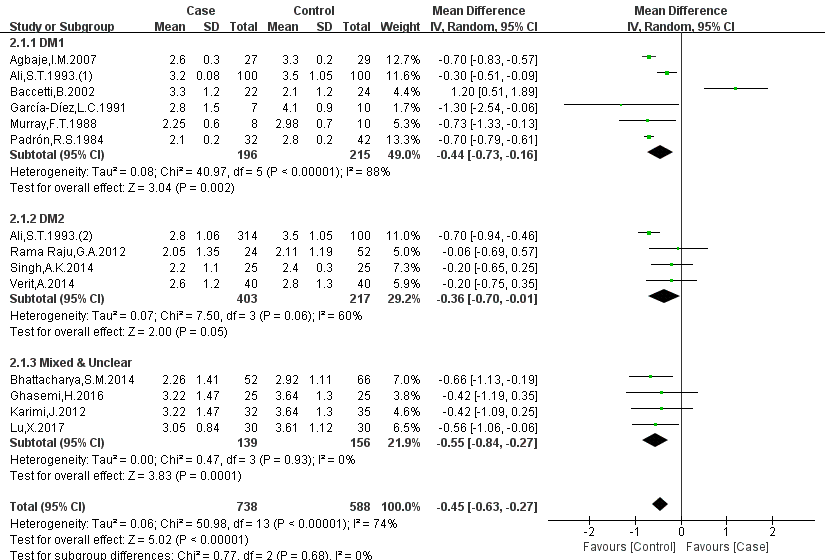


Supplemental Figure S1. Meta-analysis of the effects of different types of diabetes on semen volume.


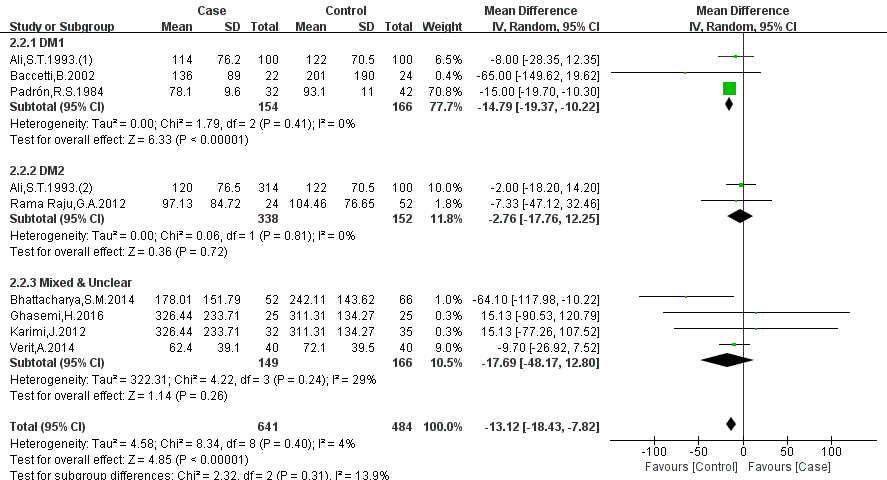


Supplemental Figure S2. Meta-analysis of the effects of different types of diabetes on semen count.


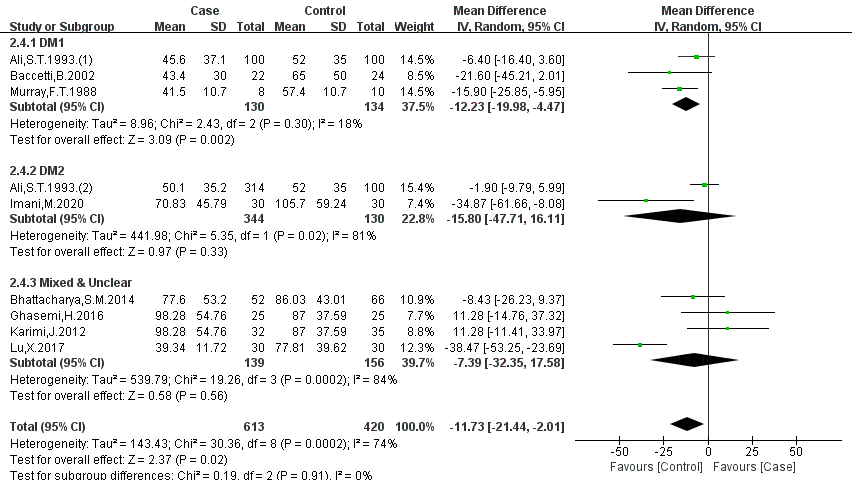


Supplemental Figure S3. Meta-analysis of the effects of different types of diabetes on semen concentration.


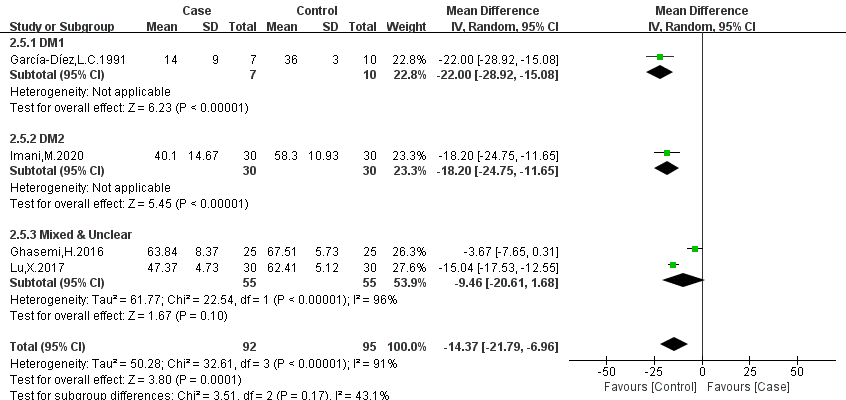


Supplemental Figure S4. Meta-analysis of the effects of different types of diabetes on progressive motility.


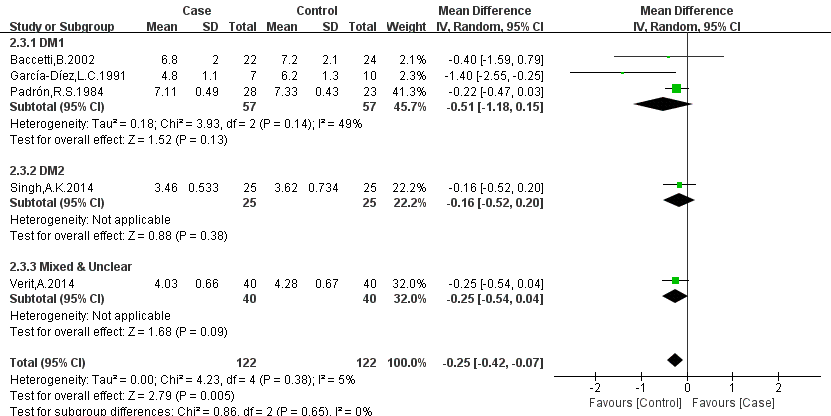


Supplemental Figure S5. Meta-analysis of the effects of different types of diabetes on testosterone levels.
